# Growth rates of malignant and benign thyroid nodules in an ultrasound follow-up study: a retrospective cohort study

**DOI:** 10.1186/s12885-019-6348-z

**Published:** 2019-11-21

**Authors:** Michael Cordes, Theresa Ida Götz, Karen Horstrup, Torsten Kuwert, Christian Schmidkonz

**Affiliations:** 1 Radiologisch-Nuklearmedizinisches Zentrum, Martin-Richter-Str. 43, 90489 Nuremberg, Germany; 2 Nuklearmedizinische Klinik, Universitätsklinikum Erlangen, Ulmenweg 18, 91054 Erlangen, Germany

**Keywords:** Thyroid nodules, Growth kinetics, Growth rates, Follow-up, Tumor growth, Thyroid carcinomas, Thyroid adenomas, Ultrasound, Differentiated thyroid carcinomas

## Abstract

**Background:**

Thyroid nodules are frequently detected by cervical ultrasound examinations. In follow-up studies, malignant as well as benign nodules may exhibit an increase in size.

The objective of our investigation was to test whether histologically determined malignant and benign thyroid nodules show differences in growth rates above a defined significance level.

**Methods:**

A retrospective ultrasound cohort follow-up study from 4 to 132 months included 26 patients with differentiated carcinomas and 26 patients with adenomas of the thyroid gland. Significance levels were determined by intra- and interobserver variations of volumetric measurements in 25 individuals.

**Results:**

Intra- and interobserver volumetric measurements were highly correlated (*r* = 0.99 and *r* = 0.98, respectively), with variations of 28 and 40%, respectively. The growth rates of malignant and benign nodules did not show differences with respect to two sonographic measurements (d = − 0.04, 95%CI(P): 0.41–0.85, *P* = 0.83). Using shorter increments and multiple measurements, growth rates of malignant nodules revealed significantly higher values (d = 0.16, 95%CI(P): 0.02–0.04, *P* = 0.039).

**Conclusions:**

The growth rates of benign and malignant thyroid nodules do not appear to differ using two sonographic volumetric measurements. However, due to temporal changes in cellular proliferation and arrest, malignant nodules may exhibit higher growth rates with multiple assessments and shorter increments.

## Background

Several articles in the medical literature suggest considering the presence of malignancy in growing thyroid nodules [1–3]. An increase in the size of a thyroid nodule, in particular, should raise concerns about its malignancy [4, 5]. Furthermore, rapid tumor growth occurring in patients who are thought to have simple nodular goiter has been acknowledged as one criterion of malignancy, especially during treatment with levothyroxine [6, 7]. In contrast, other authorities have contested this belief since benign nodules may also increase in size over time [8–10]. According to most current viewpoints, the proliferation of follicular cells seems to be inherent to benign as well as malignant tumors.

It has been pointed out that nodules with a diameter of more than 4 cm were thought to harbor a higher risk of malignancy [7]. However, others have emphasized how the size of a nodule itself could not be used to predict a benign or malignant nature [11]. In spite of this, consideration should be given that the risk of cancer is slightly higher in nodules with a diameter of more than 4 cm.

The natural course of benign thyroid nodules has been studied by Durante et al. [8]. In this paper, approximately 15% of such nodules showed continuous growth of more than 20% in a mean follow-up period of 60 months. Similar findings for the growth of benign thyroid nodules have been reported by Erdogan et al. [12]. Further evidence indicates that benign thyroid nodules may show an increase in volume, even during levothyroxine treatment [13]. However, with regard to the growth rate of malignant nodules, results have been non-uniform. One study demonstrated that a large number of papillary microcarcinomas remained relatively stable over a long observation period [14]. In contrast, in another prospective long-term investigation, more than 25% of malignant thyroid nodules showed a significant increase in size [1].

Since thyroid nodules are frequently detected by cervical ultrasound examinations, distinguishing between a benign and a malignant nodule is a relevant clinical challenge. With respect to current guidelines, it is the sonographic pattern rather than the growth of a nodule that raises suspicions of malignancy [15]. Depending on the ultrasound pattern, fine-needle aspiration biopsy (FNAB) is considered the method of choice to detect malignancy. However, some patients may refuse an FNAB or the initial FNAB result may be a false negative. Consequently, such nodules will be subject to serial follow-up ultrasound examinations.

In this retrospective cohort study, we report serial sonographic examinations in 28 malignant and 26 benign thyroid nodules selected from our clinical database and verified by histological analysis. The aim of this study was to test the hypothesis that the growth rates of benign and malignant follicular thyroid nodules, as determined by volumetric ultrasound, do not differ. For this analysis, we also took the intra- and interobserver reproducibility of volumetric ultrasound into account as determined in a subset of 25 separately studied nodules.

## Methods

This retrospective study was performed according to the principles of the Declaration of Helsinki and its subsequent amendments and according to the guidelines of the Institutional Review Board (IRB) of the Friedrich-Alexander-University, Erlangen/Nuremberg, Germany under the auspices of the Bavarian Hospital Act (Bayerisches Krankenhausgesetz Art. 27 (4)). All patients gave general permission for the use of their clinical data for scientific purposes and written informed consent for the anonymous publication of data.

### Inclusion criteria

In this single center study we included 77 patients with thyroid nodules (age range: 22 to 83 years) with their biographic data shown in Table 1. Demographic, sonographic and pathologic information were obtained from our database. We reviewed patients who were examined between 2008 and 2018.

**Table 1:** Biographic data of the patients

	All patients	Subset A (intra-interobserver group)	Subset B (carcinoma group)	Subset C (adenoma group)
Number of patients	77	25	26	26
Mean age [y]	51.6	54.4	50.3	50.1
SD [y]	14.1	12.7	15.0	14.1
Range [y]	22–83	22–83	22–75	24–75

The age of the patients is given in years (y) at initial presentation. Statistically there was no difference of the mean age in the three subsets A (intra−/interobserver variation group), B (carcinoma group) and C (adenoma group) (*P* = 0.48, ANOVA). SD: standard deviation

From our database we could identify 25 patients with thyroid nodules who were assessed with respect to intra- and interobserver variations of sonographic volumetry. This assessment had been carried out for quality assurance purposes in our institution (subset A: intra−/interobserver variation group).

In our database 52 patients were documented who had serial ultrasound examinations of thyroid nodules.) At the end of the follow-up period the thyroid nodules represented histologically confirmed differentiated thyroid carcinomas in 26 patients (subset B: carcinoma group) and histologically confirmed follicular adenomas in 26 patients (subset C: adenoma group, respectively. The pathologic examinations were carried out by two board certified pathologists with special expertise of at least 10 years in thyroid tumors. Only patients with definite histologic diagnoses were includes in our study. Patients with questionable histologic diagnoses were not considered for our evaluation.

### Ultrasound examinations

For sonographic examinations of the neck region, we used two ultrasound devices: Logiq P6 Pro (D1); General Electric, Chicago, IL, USA, and X6, Sono Ace (D2); Samsung Healthcare, Seoul, South Korea. The ultrasound devices were equipped with high resolution longitudinal probes operating at transmitting frequencies of 10.0 and 10.3 MHz, respectively. Sonographic examinations were performed by three board-certified nuclear medicine physicians (P1, P2 and P3). Every physician had an experience of at least 10 years in thyroid sonography. Standardized examination protocols including transverse and longitudinal slice orientations were used for thyroid sonography. All nodules were classified according to the American Thyroid Association classification system [15] with respect to their risk of malignancy: type 1: benign; type 2: very low risk; type 3: low risk; type 4: intermediate risk and type 5: high risk.

The size of the thyroid nodules was measured in three dimensions (dx, dy, and dz) using the internal calipers of the devices, where dx, dy, and dz represented the diameter in transverse, sagittal, and longitudinal directions, respectively. The volume (vol) of the nodules was calculated as $\mathrm{vol}=\frac{\left(\mathrm{dx}\bullet \mathrm{dy}\bullet \mathrm{dz}\right)}{2}$. For follow-up examinations, the percentage of volume change (dvol%) per month was expressed according to $\frac{\mathrm{dvol}\%}{\mathrm{dt}}=\frac{\left({\mathrm{vol}}_{\mathrm{t}}-{\mathrm{vol}}_{\mathrm{t}0}\right)\bullet 100\%}{{\mathrm{vol}}_{\mathrm{t}0}\bullet \left(\mathrm{t}-{\mathrm{t}}_0\right)}$, where t_0_ and t represented the time points of the first and follow-up examinations, respectively.

#### Ultrasound examinations in subset A

These patients were examined using sonography by two physicians (P1 and P2) who were blinded to each other at one time point using the two ultrasound devices, D1 or D2. Measurements by P1 using D1 were used as the reference values. Measurements by P1 using D2 were correlated with the reference values to calculate intraobserver variation, and measurements by P2 using D2 were used to calculate interobserver variation.

#### Ultrasound examinations in subset B and C

Every ultrasound examination in subset B and C were performed by one of the physicians P1, P2 or P3. All patients of these subsets had one initial thyroid ultrasound scan (baseline examination at time point t_0_) and follow-up examinations at different time points t. The growth rates of the thyroid nodules were calculated with respect to the baseline examination at time point t_0_ and the final examination at time point T (“Dual Recordings”). In addition, the growth rates of the thyroid nodules were also calculated for shorter increments when ultrasound measurements were carried out at time point t between t_0_ and T (“Multiple Recordings”).

Subset B included 26 patients with 28 malignant nodules (age range: 22 to 75 years at initial examination). Seven (25%) of the 28 nodules were classified as type 2 or 3, and 21 (75%) of the 28 as type 4 or 5 using sonography. The time interval between initial examination and the final follow-up examination at time point T was between 4 and 132 months. Nine of the 26 patients had additional ultrasound examinations between t_0_ and T at time point t. The median time between t_0_ and T was 29.5 months (range 4 to 132 months), and between t_0_ and t it was 26 months. A total number of 71 ultrasound examinations was carried out in this subset of patients. All patients had a thyroidectomy and the malignant nature of the thyroid nodule was determined histologically. Twenty-four of the nodules were classified as papillary thyroid carcinomas (PTCs) and four nodules as follicular thyroid carcinomas (FTCs).

Subset C included 26 patients with 26 benign thyroid nodules (age range: 24 to 75 years at initial presentation). Eighteen (69%) of the 26 nodules were classified as type 2 or 3, and 8 (31%) of the 26 as type 4 or 5 using sonography. The time interval between the initial presentation and the final follow-up examination was between 7 and 93 months. Nineteen of the 26 patients had additional ultrasound examinations between t_0_ and T at time point t. The median time between t_0_ and T was 52 months (range 7 to 93 months) and between t_0_ and t it was 42 months (range 3 to 93 months). A total number of 87 ultrasound examinations were carried out in this subset of patients. All patients had a thyroidectomy and the benign nature of the thyroid nodules was confirmed histologically. Five of the nodules were classified as microfollicular, 12 nodules as macrofollicular and nine nodules as mixed follicular thyroid adenomas.

All patients’ data were de-identified before the analyses were done. Statistical analyses were performed using Winstat^R^ version 2012.1.0.96 and MATLAB version R2012b. Continuous parameters are given as mean ± standard deviation (SD). Multiple comparisons among observers were performed by repeated measures ANOVA with post hoc Bonferroni corrections and calculations of F values. The intra- and interobserver differences are presented as the mean difference ± standard error (SE). Differences between two groups were evaluated by using a Wilcoxon rank-sum test. Differences between frequency parameters were tested using the chi-square test. The relationship between values determined by different observers was analyzed by Pearson’s product-moment correlation coefficient (r). For all analyses, a *P*-value <0.05 was considered significant.

## Results

### Baseline examinations of all subsets

The mean volume, standard deviation, and minimum and maximum values of all nodules in subsets A, B, and C were 2.5 mL, 4.2 mL, 0.1 mL, and 27.1 mL, respectively. Differences in the thyroid nodule volume among the reference values in subset A and in the initial measurements in subsets B and C were not noted (d = 0.3051, 95%CI(d): − 0.2318 – 0.842, *P* = 0.53, ANOVA).

### Subset a: intra- and Interobserver variation

Intraobserver variation: The nodule volumes determined by baseline measurements made by P1 showed a high correlation with reference values (r = 0.99 Pearson’s product-moment correlation). The variation of the percentage volume difference $\mathrm{dvol}\%=\frac{\left({\mathrm{vol}}_2-{\mathrm{vol}}_1\right)\bullet 100\%}{{\mathrm{vol}}_1}$, expressed as +/− 2 standard deviations (SD), was 28% where vol1 represented the reference value and vol2 was the repeated measurement made by P1.

Interobserver variation: The nodule volumes determined from measurements made by P1 and P2 demonstrated a correlation of *r* = 0.98 (*P* < 0.001; Pearson’s product-moment correlation). The variation of dvol% was expressed as ±2 SD and was found to be 40%.

### Subsets B and C: growth rates of malignant and benign nodules

Nodule volumes increased significantly between t_0_ and T in subsets B and C (d =  − 0.21, 95 % CI(P) : 0.00 − 0.12, *P* < 0.001 and d =  − 0.21, 95 % CI(P) : 0.00 − 0.12, *P* < 0.001 , respectively, Wilcoxon test). Statistically, a difference in nodule volume between subsets B and C at t_0_ and T was not noted (d =  − 0.07, 95 % CI(P) : 0.42 − 0.87, *P* = 0.81 and d = 0.12, 95%CI(P): 0.46–0.94, *P* = 0.93, respectively, Wilcoxon test). The values of the nodule volumes are shown in Table 2.

**Table 2:**
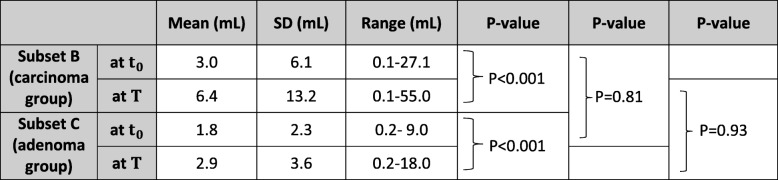
Statistical analysis (Wilcoxon Test) of the nodule volume for subsets B (carcinoma group) and C (adenoma group) at baseline (t_0_) and the final follow-up examinations (T). The median follow-up times were 29.5 and 52 months in subset B and subset C, respectively

dvol % /dt was calculated for subsets B and C. Two statistical approaches were performed: In the first approach, measurements at time points t_0_ and T were taken into account, and in approach two, measurements at all time points were taken into account.

#### Approach 1 (“Dual Recordings”).

**Fig. 1:**
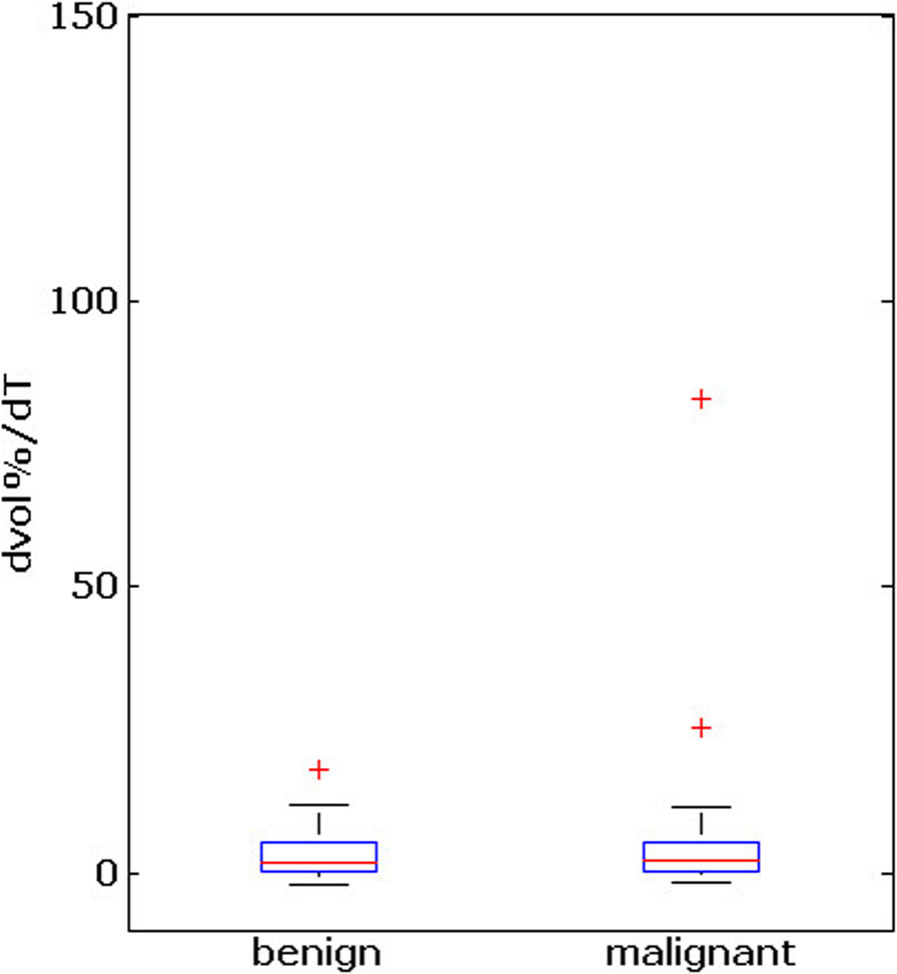
Box plots of the growth rates of malignant (subset B) and benign (subset C) thyroid nodules between time points t0 and T (approach 1). The whiskers indicate 1.5 interquartile ranges above and below the lower and and upper quartiles of the data

With respect to baseline measurements and final follow-up examinations, the growth rate in malignant and benign thyroid nodules was not statistically different (d =  − 0.04, 95 % CI(P) : 0.41 − 0.85, *P* = 0.83, Wilcoxon test; see Fig. 1).

#### Approach 2 (“Multiple Recordings”).

**Fig. 2:**
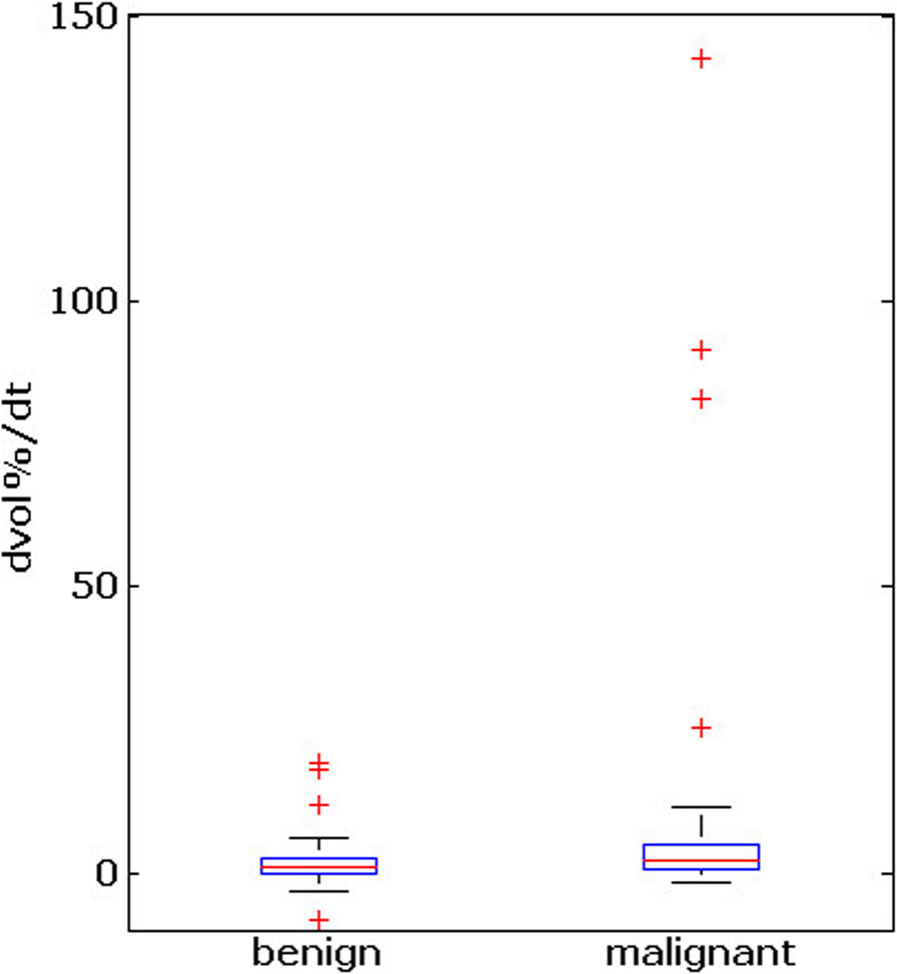
Box plots of the growth rates of malignant (subset B) and benign (subset C) thyroid nodules among all time points t0 and t (approach 2). The whiskers indicate 1.5 interquartile ranges above and below the lower and and upper quartiles of the data

The growth rates of malignant nodules in subset B and benign nodules in subset C were calculated, including measurements at all time points each referred to the first measurement. The growth rate in malignant and benign thyroid nodules was statistically different (d = 0.16, 95 % CI(P) : 0.02 − 0.04, *P* = 0.039, Wilcoxon test; see Fig. 2).

The growth rates of nodules were also tested for subgroups, gender, age <50 vs. ≥50 years and ultrasound appearance within subsets B and C using the two approaches. Both approaches did not reveal different growth rates for malignant and benign nodules.

The values for the growth rates of subsets B and C as well as the subgroups are shown in Tables 3 and 4.

**Table 3:** Values of the growth rates (dvol % /dT) for the subgroups in subsets B (carcinoma group) and C (adenoma group) using the statistical approach 1 with a median follow-up time of 29.5 and 52 months, respectively. us: ultrasound

Approach 1 [„Dual Recordings]				
	Mean [%/months]	SD [%/months]	Range [%/months]	*P*-value
subset B (carcinoma group)				
female (*n* = 22)	6.7	17.5	−1.6 - 82.8	0.125
male (*n* = 4)	4.9	2.3	2.0–7.3	
age < 50 years (*n* = 14)	9.8	22.1	−1.6 - 82.8	0.827
age ≥ 50 years (*n* = 12)	2.9	2.9	−0.7 - 11.3	
us type 2 or 3 (*n* = 7)	2.8	2.5	0.1–7.3	0.979
us type 4 or 5 (*n* = 21)	7.0	17.4	−1.6 - 82.8	
subset C (adenoma group)				
female (*n* = 22)	3.4	3.96	−2.0 - 12.0	0.525
male (*n* = 4)	7.5	9.54	−0.3 - 18.2	
age < 50 years (*n* = 12)	3.4	3.44	− 0.7 - 11.3	0.360
age ≥ 50 years (*n* = 14)	3.9	5.14	− 2.0 - 18.2	
us type 2 or 3 (*n* = 18)	3.4	3.98	−0.3 - 12.0	0.902
us type 4 or 5 (*n* = 8)	4.0	5.33	−2.0 - 18.2

**Table 4:** Values of the growth rates (dvol %/dt) for the subgroups in subsets B (carcinoma group) and C (adenoma group) using the statistical approach 2 with a median follow-up time of 26 and 42 months, respectively. us: ultrasound

Approach 2 [„Multiple Recordings“]				
	Mean [%/months]	SD [%/months]	Range [%/months]	*P*-value
subset B (carcinoma group)				
female (*n* = 22)	10.7	29.1	−1.6 - 142.4	0.117
male (*n* = 4)	4.9	2.3	2.0–7.3	
age < 50 years (*n* = 14)	19.3	39.1	−1.6 - 142.4	0.361
age ≥ 50 years (*n* = 12)	1.8	2.1	−2.0 - 7.3	
us type 2 or 3 (*n* = 7)	2.2	2.5	−1.5 - 7.3	0.57
us type 4 or 5 (*n* = 21)	8.1	24.8	−1.6 - 142.4	
subset C (adenoma group)				
female (*n* = 22)	1.8	4.0	−8.3 - 19.3	0.337
male (*n* = 4)	4.5	7.0	−0.3 - 18.2	
vage <50 years (*n* = 12)	2.3	4.1	−1.6 - 19.3	0.960
age ≥ 50 years (*n* = 14)	1.7	3.9	−8.3 -18.2	
us type 2 or 3 (*n* = 18)	2.1	4.2	−8.3 - 19.3	0.088
us type 4 or 5 (*n* = 8)	1.8	4.0	−2.0 - 18.2	

### Nodules with volume changes above the interobserver variation

Eleven of 28 (39%) malignant and 14 of 26 (54%) benign nodules demonstrated an increase in volume above the interobserver variation (d = 0.8829, 95%CI(d): 0.2998–1.4660, *P* = 0.003, Chi-square test). This volume increase was seen in 9 of 19 (47%) of patients <50 years and in 2 of 9 (22%) of patients ≥50 years of age with malignant nodules (d = 1.5881, 95 % CI(d) : 0.6422 − 2.534, *P* < 0.001, Chi-square test), as well as in 13 of 18 (72%) of patients <50 years and in 6 of 8 (75%) of patients ≥50 years of age with benign nodules (d = 0.2733, 95 % CI(d) :  − 0.5026 − 1.0492, *P* = 0.49, Chi-square test).

With respect to their histological classification, 9 of 24 (38%) of PTCs and 2 of 4 (50%) of FTCs showed a volume increase of more than 40% (d = 1.0226, 95 % CI(d) : 0.1906 − 1.8546, *P* = 0.016, Chi-square test).

In nodules classified by sonography as type 2 or 3, this enlargement was detected in 1 of 7 (14%) malignant and in 10 of 18 (55%) benign tumors (d = 1.7481, 95 % CI(d) : 0.7069 − 2.7894, *P* < 0.001, Chi-square test). In contrast, nodules classified as type 4 or 5 with this feature were diagnosed as malignant in 10 of 21 (48%) and benign in 4 of 8 (50%) cases (d = 0.1485, 95%CI(d): − 0.5814 – 0.8785, *P* = 0.69, Chi-square test).

## Discussion

In this study, we found no significant differences in the growth rates of malignant and benign thyroid nodules, thus providing a linear model of nodule enlargement. The kinetics of thyroid nodule growth have been subjected to several other investigations. In a previous report by Asanuma et al. tumor growth did not differ between malignant and benign nodules [16]. However, in contrast to our study only PTCs versus benign neoplasms were evaluated and their follow-up time did not exceed 85 months for both entities.

In our study the growth rates of only 4 FTCs were analyzed. Therefore, we did not test the tumor growth rates of FTCs versus PTCs. In a recent study, Kim et al. compared the growth rates of follicular adenomas and follicular carcinomas of the thyroid gland by serial ultrasound measurements [17]. In their retrospective study 50 FTCs and 110 follicular adenomas were included. After a median follow-up time of 4 years, no significant difference in growth rates was found between these two entities.

The assumption of a linear progression may not reflect the real growth kinetics of neoplastic tissue. In contrast to this linear model, we found that shorter increments of multiple volume determinations revealed differences in the growth rates between malignant and benign thyroid tumors.

By using multiple volume measurements at shorter increments compared with only two recordings at the initial and final time points, we identified growth rates of malignant nodules that were higher than those of benign tumors. However, the evidence for this finding in our study is limited since we did not perform ultrasound examinations at shorter increments in all patients. Tuttle et al. performed serial multiple ultrasound measurements in more than 290 patients with PTCs undergoing active surveillance at a tertiary referral center [14]. During a median follow-up period of 25 months they found that 12% of PTCs demonstrated a volume increase of more than 50%. These tumors demonstrated exponential growth patterns which underline the importance of serial multiple volumetric recordings. The other 88% of neoplasms remained stable with respect to their volume. These finding are in accordance with the 14% of differentiated carcinomas in our study which showed a volume increase of more than 40%.

The tumor proliferation and arrest as well as the tumor growth patterns might be due to the somatic mutations that have been identified in benign and malignant thyroid neoplasms [18]. In follicular adenomas, point mutations of RAS and other genes have been disclosed. Similar mutations have also been found in FTCs, whereas PTCs often show mutations of RET/PTC and BRAF oncogenes [19]. Oncogene actions cause cell proliferation and tumor growth mediated by their protein products. However, current thinking is that oncogenetic actions also induce cell senescence resulting in apoptosis [20]. It has been acknowledged that the carcinogenesis process involves genetic and metabolic transformations leading to malignant cells derived from normal precursors. With respect to the kinetics of malignant tissues, periods of growth and proliferation as well as periods of arrest have been postulated [21]. In this context, periods of arrest may allow the metabolic refueling of malignant cells.

For the assessment of the nodules’ volume the diagnostic accuracy of ultrasound volumetry has to be taken into account. In this context, the intra- and interobserver variations of ultrasound measurements represent inherent constraints of follow-up examinations. In our retrospective study, volume measurements were carried out by three physicians. We considered a volume increase of more than 40% as significant. This interobserver variation of 40% is in close agreement with the previously reported data of Brauer et al. who found an interobserver variation of 49% for the sonographic volumetry of thyroid nodules [22].

### Limitations

The investigation that we carried out has several limitations. The retrospective study design may have caused significant bias of the data. We included only patients in our study who were surgically treated and in whom the histological results were available. Therefore, we did not record the natural course of non-operated malignant and benign thyroid nodules. Except for the interobserver measurements, each thyroid ultrasound examination was carried out by only one physician. In total, three physicians were involved in the ultrasound studies of all patients. The results of these three physicians were not cross-checked and thus not validated. However, we are confident that the variation of volume assessments among these experienced examiners is acceptably low according to the high correlation as demonstrated by the intra- and interobserver measurements. Confounding factors may also be the small number of the patients and the non-uniform, and in some cases short intervals of the follow-up examinations. Therefore, the results of our study should be interpreted with caution since confounding and bias errors due to the nature of our retrospective study design may not be excluded.

## Conclusions

The growth rate of benign and malignant thyroid nodules do not appear to differ when using sonographic volumetric measurements at two time points. However, due to temporal changes in cellular proliferation and arrest, malignant nodules may exhibit higher growth rates with multiple assessments and shorter increments.

## Data Availability

The datasets used and/or analyzed during the current study are available from the corresponding author on reasonable request.

## Abbreviations

95%CI(d): 95% confidence interval for d
95%CI(P): 95% confidence interval for P
BRAF: Rapidly accelerated fibrosarcoma protein b
d: Cohen’s d effect size
dvol%: Percentage of volume change
FNAB: Fine-needle aspiration biopsy
FTC: Follicular thyroid carcinoma
PTC: Papillary thyroid carcinoma
RAS: Rat sarcoma protein
RET/PTC: Receptor tyrosine kinase/ papillary thyroid carcinoma
vol: Volume
